# Bulk versus Contact Doping in Organic Semiconductors

**DOI:** 10.3390/mi12070742

**Published:** 2021-06-24

**Authors:** Chang-Hyun Kim

**Affiliations:** Department of Electronic Engineering, Gachon University, Seongnam 13120, Korea; chang-hyun.kim@gachon.ac.kr

**Keywords:** organic semiconductors, field-effect transistors, doping, contact effects

## Abstract

This study presents a comparative theoretical analysis of different doping schemes in organic semiconductor devices. Especially, an in-depth investigation into bulk and contact doping methods is conducted, focusing on their direct impact on the terminal characteristics of field-effect transistors. We use experimental data from a high-performance undoped organic transistor to prepare a base simulation framework and carry out a series of predictive simulations with various position- and density-dependent doping conditions. Bulk doping is shown to offer an overall effective current modulation, while contact doping proves to be rather useful to overcome high-barrier contacts. We additionally demonstrate the concept of selective channel doping as an alternative and establish a critical understanding of device performances associated with the key electrostatic features dictated by interfaces and applied voltages.

## 1. Introduction

Organic semiconductors are enabling a new area of flexible electronics by virtue of their molecular-level softness and competitive low-temperature processabilities [1,2,3,4,5]. After the successful commercialization of organic light-emitting diodes (OLEDs), other types of organic electronic devices are now poised to conquer the market, emphasizing the same merits of high multi-functionality, unconventional physical form factors, and additive, printing-based customizable manufacturing. The organic field-effect transistor (OFET) is one such emerging candidate [6,7,8,9,10,11,12,13] that can play a key role in active-matrix displays, image sensors, and bio-neuromorphic systems.

Despite the dramatic performance improvements made for OFETs over the past few decades, there is still a lack of fundamental understanding regarding their physical aspects. For instance, charge doping, one of the most established design and engineering concepts for traditional (inorganic) semiconductor devices, still requires more understanding because the nature and manifestation of doping in organic materials significantly differ from those in inorganic counterparts [14,15,16]. In particular, the vast majority of reported organic devices are not (nominally) doped at all, yet native or unintentional doping can always take place under realistic conditions [17]. This makes it even more unclear whether a dedicated doping step is really necessary for organic semiconductors. Moreover, the vastly different doping methods, ranging from chemical solution mixing to physical diffusion (or modulation) doping, as well as various possible focus zones inside an OFET device structure, are among the major complications.

In this study, we address this difficult but important issue of OFET doping by drawing systematical theoretical comparisons between several doping motifs relevant to state-of-the-art OFET devices. We especially focus on comparing bulk and contact doping, both of which have been frequently adopted in the literature. Considering the specificities of the topic, we take the maximum leverage of the structural device simulation in precisely controlling the doping locations and densities. Furthermore, both co-doping and counter-doping scenarios are fairly considered, the latter of which has not been adequately addressed in organic semiconductors.

## 2. Experimental and Simulation Methods

Flexible OFETs were fabricated as a model device platform to be reproduced in theoretical simulation. These devices are of a staggered-type, and have a bottom-gate and top-contact configuration [18]. A polyethylene terephthalate (PET) plastic was used as a substrate. Indium-tin oxide (ITO) covered with a poly(3,4-ethylenedioxythiophene):poly(styrenesulfonate) (PEDOT:PSS) buffer was used a double-layer gate electrode. The ITO layer was pre-deposited upon purchase of the PET substrates (Sigma-Aldrich, Saint Louis, MO, USA). The PEDOT:PSS film was deposited by spin coating at 3000 rpm and annealing at 100 °C. A 410-nm-thick polymeric dielectric film was formed by spin coating a poly(methyl methacrylate) (PMMA, M.W. = 120 k, Sigma-Aldrich) solution in toluene (with a concentration of 60 mg/mL) at 2000 rpm. The as-deposited PMMA film was immediately annealed at 100 °C. A 30-nm-thick *p*-type organic semiconductor film was deposited by thermal vacuum evaporation of dinaphtho[2,3-b:2′,3′-f]thieno[3,2-b]thiophene (DNTT, sublimed grade, 99%, Sigma-Aldrich) through a shadow mask [19,20,21]. The device fabrication was finished by thermal vacuum evaporation of Au source and drain electrodes through another shadow mask defining the channel width (*W*) and length (*L*) of 500 and 50 μm, respectively. The electrical characteristics of the OFETs were recorded using a Keithley 4200 semiconductor parameter analyzer in the dark and under ambient conditions.

As a simulation tool, a two-dimensional (2D) finite-element simulation package (ATLAS, Silvaco, USA) was employed. This is a physically based numerical simulator widely used for device research [22,23,24,25,26,27,28,29]. Here, we briefly explain its core capabilities. Poisson’s equation relates the local potential to the space charge density as

(1) div(ε∇ψ)=−ρ

where *ψ* is the electrostatic potential, *ε* is the semiconductor permittivity, and *ρ* is the local space charge density. In addition, the drift-diffusion transport model is given as

(2) $$\vec{J_{n}}=qn\mu_{n}\vec{E_{n}}+qD_{n}\nabla n$$

and

(3) $$\vec{J_{p}}=qp\mu_{p}\vec{E_{p}}-qD_{p}\nabla p$$

where $\vec{J_{n}}$ and $\vec{J_{p}}$ are the electron and hole current density, respectively; $\vec{E_{n}}$ and $\vec{E_{p}}$ are the electron and hole effective electric field, respectively; *μ_n_* and *μ_p_* are the electron and hole mobility, respectively; *D_n_* and *D_p_* are the electron and hole diffusion coefficient, respectively; *q* is the elementary charge; and *n* and *p* are the electron and hole concentration, respectively. The simulator solves these Poisson’s and drift-diffusion equations in a self-consistent manner for an entire 2D mesh structure that is user-defined to model or predict an experimental device. Note that the charge-carrier densities (*n* and *p*) and associated *ρ* are locally determined from the intrinsic carrier density and doped carrier density contributions, so that we can have direct control over these values and their gradients in Equations (1)–(3) (which we will strategically use for the doping study). An application of external electrical biases to the device alters the overall potential landscape, which in turn affects the field vectors as well as the 2D carrier distribution. Therefore, solving Equations (1)–(3) under a stated bias condition provides access to the current density ($\vec{J_{n}}$ and $\vec{J_{p}}$) generated due to this biasing. The terminal currents (or macroscopic currents measurable by test equipment) are simply estimated by integrating local current densities over the whole 2D mesh (or device) and *W*. Therefore, this type of numerical solver is able to produce practical current–voltage curves of the device (e.g., transfer curves for transistors) by repeating this procedure over a certain voltage sweep range.

## 3. Results and Discussion

### 3.1. Validity of Simulation Framework

**Figure 1:**
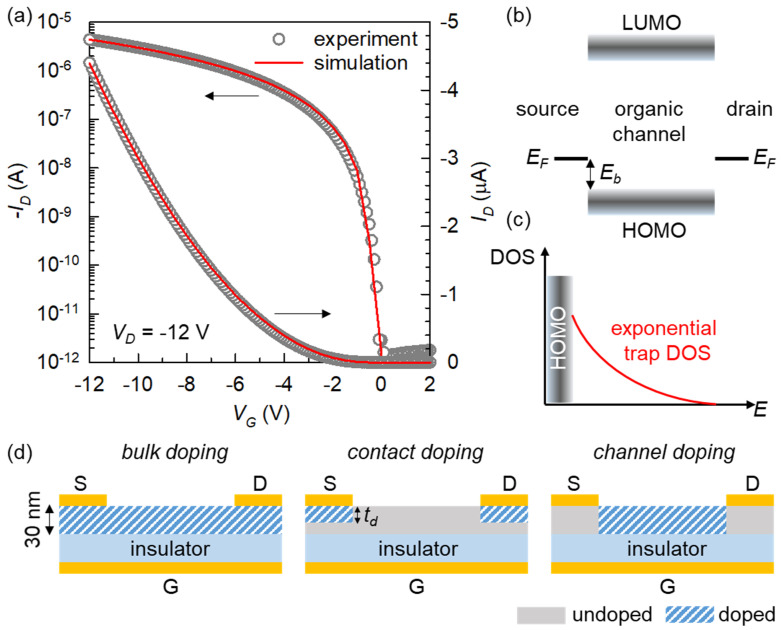
(**a**) Comparison between the experimental and simulation-optimized OFET transfer characteristics. (**b**) Energy levels related to the source-to-drain conduction. (**c**) Trap DOS distribution. (**d**) Structural differences between the bulk, contact, and channel doping methods: G, gate; S, source; D, drain.

We first set up a base simulation framework that is able to closely reproduce the experimental OFET. This task involved a series of careful optimization steps for finding the optimum materials and interface input parameters. The excellent agreement between the gate voltage (*V_G_*) versus drain current (*I_D_*) characteristics (or transfer characteristics) at a drain voltage (*V_D_*) of −12 V from the measurement and numerical simulation in Figure 1a broadly supports the validity of the model and parameters used. We specifically took into account several unique properties of OFETs, including the effect of a charge injection barrier (*E_b_*) at the metal–semiconductor contact (Figure 1b) [30,31,32] and a trap- or disorder-limited transport mediated by an exponential density of states (DOS) (Figure 1c) [33,34,35]. The optimized parameters are listed in Table 1. The zero-doping condition, which usually represents a very low thermal charge-carrier density in an unintentionally doped semiconductor [17], is assumed here. In this context, Figure 1a is good evidence that such a nominally intrinsic organic channel can fully realize a working field-effect device, while the following results will argue about the applicable range of such a simplistic assumption.

Figure 1d illustrates the concepts of the three different doping techniques. Experimentally, bulk doping can be obtained by exposing a pristine organic semiconductor film to doping agents (usually in liquid or gas phase) or by forming a co-solution of a host-dopant mixture that is directly processed (e.g., by spin coating) to become a doped composite semiconductor layer [36,37,38]. Contact doping has also been widely adopted, as it is fundamentally associated with a generally high contact resistance of OFETs [32]. This type of doping can be experimentally achieved, for instance, by shadow masking upon thermal evaporation of dopant molecules, or by direct co-patterning of the blanket-deposited dopant and metal contact layers [39,40,41,42]. As a third method, channel doping has been less frequently reported. It was shown that a practical channel doping step may include thermal dopant evaporation on a pre-deposited source and drain contacts serving as a self-alignment mask or direct solution-phase dopant printing over selective channel areas [43,44].

It is important to note that having an experimentally validated base framework (Figure 1a along with Table 1), with the parameter set carefully optimized against the measured trend, strongly supports the assumption of an extremely low intrinsic doping level in usual OFETs. More importantly, it also allows for a robust predictive comparison between any structural and material modifications by, for instance, position- and density-dependent doping methods investigated hereinafter. On the other hand, it is worth clarifying that, due to practical materials and processing limitations, it is extremely challenging to experimentally realize and compare all the different doping schemes on sufficiently common ground. Therefore, the main goal of this study is to draw a fair theoretical comparison focusing on fundamental structural aspects, by utilizing exactly the same simulation base structure and by selecting and introducing systematic doping parameters that are relevant to all types of doping.

### 3.2. Bulk Doping

**Figure 2:**
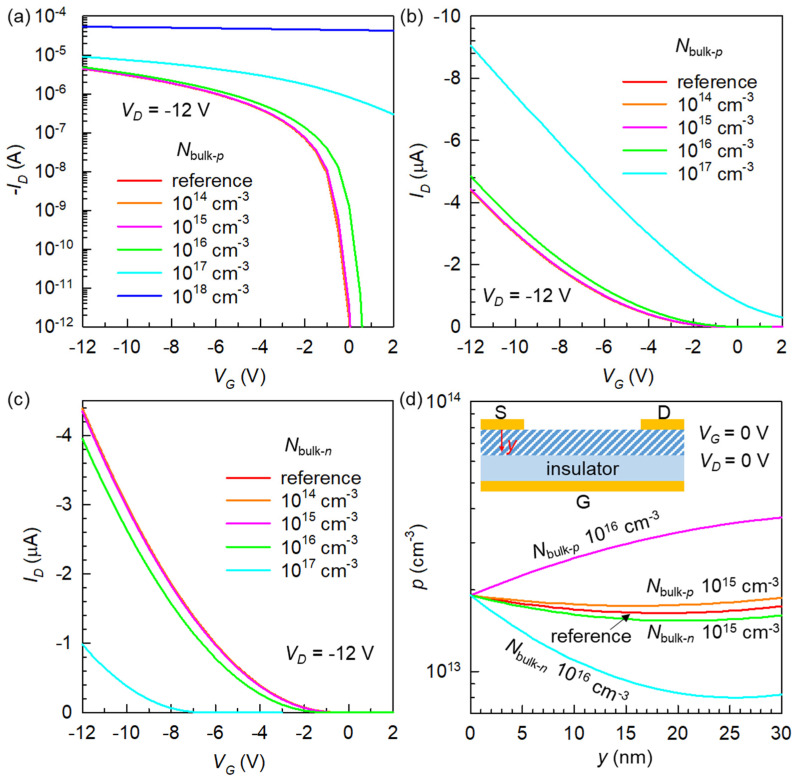
(**a**) Log-scale and (**b**) linear-scale transfer curves showing the effect of *p*-type bulk doping. (**c**) Linear-scale transfer curves showing the effect of counter (*n*-type) doping. (**d**) Hole-density profile inside the organic semiconductor below the source electrode under different doping conditions and without electrical biases. Inset: illustration of the position *y*-axis.

To verify the effect of the bulk doping level on the transistors, a *p*-type bulk doping density (*N*_bulk-*p*_), from 10^14^ to 10^18^ cm^−3^, was added to the undoped reference device. The log-scale saturation regime transfer curve in Figure 2a demonstrates that an *N*_bulk-*p*_ up to 10^15^ cm^−3^ has only a marginal impact on *I_D_*. A further increase in concentration causes a substantial positive shift in threshold voltage (*V_T_*), along with an overall increase in *I_D_* (in magnitude). This observation, first of all, directly confirms the fact that any unintentional doping during fabrication or storage (which can be described as bulk doping) up to 10^15^ cm^−3^, would not significantly affect the overall switching characteristics, since OFETs heavily rely on injected carriers [45]. Figure 2b presents the same data set on a linear scale. The case of *N*_bulk-*p*_ = 10^18^ cm^−3^ was excluded here because that device could not be turned off by a realistically high positive *V_G_*. This plot clearly shows that well-controlled doping in the 10^16^ to 10^17^ cm^−3^ range can be ideal to achieve an improved device with both large currents and effective switching.

As a next step, an *n*-type bulk doping density (*N*_bulk-*n*_) was added to the reference OFET. For simplicity, we consider this as a type of counter doping, while its exact meaning differs fundamentally from that in traditional semiconductor terminologies. In traditional semiconductors, a majority carrier type (*p*- or *n*-type) is determined by material doping (e.g., during wafer production), and counter doping (or compensation doping) implies the use of opposite-polarity dopants for reducing the amount of existing majority carriers or for implanting a local type-inverted zone. However, the conduction carrier type in an OFET is largely dictated by an energetic alignment at the metal–semiconductor interface (Figure 1b), and whether the metal Fermi level (*E_F_*) approaches the highest occupied molecular orbital (HOMO) or lowest unoccupied molecular orbital (LUMO) of the given organic molecule becomes a primary factor [46]. Our reference device (even with zero doping) exhibited an apparent *p*-type field-effect behavior because the hole *E_b_* of 0.4 eV is much lower than the electron *E_b_* of 2.9 eV (Table 1). With this in mind, we investigate an unusual case of counter doping in OFETs to assess its potential applicability. Figure 2c shows that, similar to *p* doping, the *N*_bulk-*n*_ up to 10^15^ cm^−3^ did not have a meaningful effect. However, larger doping densities were able to result in a substantial negative *V_T_* shift and systematic *I_D_* decrease. The condition of *N*_bulk-*n*_ = 10^18^ cm^−3^ was also simulated, but the result was not included because the simulator could not reach a numerical convergence, most likely due to too low currents. Despite this technical limit, we can theoretically infer that a high *N*_bulk-*n*_ (even in excess of 10^18^ cm^−3^) would not be able to cause a total polarity inversion (to an *n*-type OFET) because of the presence of strong electron-blocking contacts.

Another usefulness of a structural finite-element method lies in its ability to look inside a functional device. To further our understanding of doped transistors, we traced *p* over the physical region between the source electrode and gate insulator under both co-doped and counter-doped conditions. The data in Figure 2d reveal one important feature about charge doping in OFETs; whether it is *p*- or *n*-type doping, and no matter how large the doping level is, the holes there have an actual density much lower than the input *N*_bulk-*p*_ or *N*_bulk-*n*_ value. This should be understood as a direct outcome of a substantial *E_b_* (0.4 eV), which creates a strong depletion force over the vertical region below the metallurgical contact. Nonetheless, we also recognize that an introduced bulk doping has a capacity to further reduce (in the case of *N*_bulk-*p*_) or enhance (in the case of *N*_bulk-*n*_) the charge depletion effect at this region in modulating the overall current flow.

### 3.3. Contact Doping

**Figure 3:**
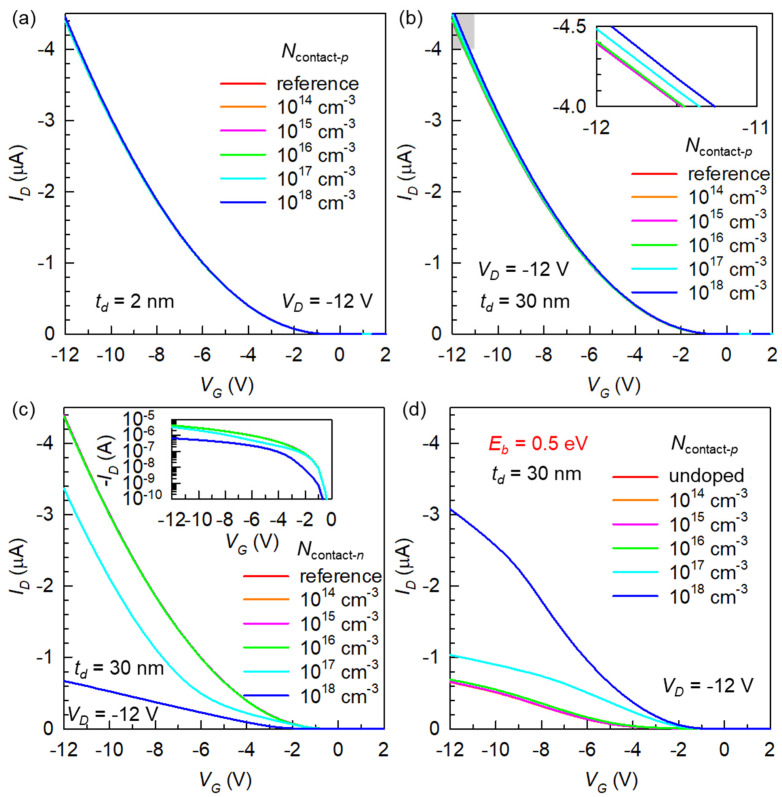
Effects of the *p*-type contact doping at (**a**) *t_d_* = 2 nm and (**b**) *t_d_* = 30 nm. The inset to (**b**) is a magnified view of the highlighted region. (**c**) Effects of the *n*-type contact doping at *t_d_* = 30 nm. Inset: the same results presented on a log scale. (**d**) Verification of the *p*-type contact doping effect on a different base OFET with an increased *E_b_* of 0.5 eV.

A contact doping technique may have a surface-localized nature or can have a rather diffusive characteristic. Therefore, we define the doping thickness (*t_d_*), as shown in Figure 1d, to observe its possible impact. In the case of shallow doping (*t_d_* = 2 nm), we found that there is no substantial change in device performance for any *p*-type doping concentration (*N*_contact-*p*_) considered (Figure 3a). This can be associated with the strong contact depletion discussed above, in that it practically deactivates even nominally high-concentration doping. Full-thickness doping (*t_d_* = 30 nm) could eventually show some effect of *N*_contact-*p*_, as shown in Figure 3b, while it was not comparable to that of bulk doping. The OFET characteristics with an *n*-type contact doping concentration (*N*_contact-*n*_) at *t_d_* = 30 nm are provided in Figure 3c. Compared to bulk doping, here, an elevated counter doping up to 10^18^ cm^−3^ could be inserted. It was found that, even with the spatial localization of dopants, this type of counter doping was efficient in decreasing the overall *I_D_*, which we believe might be useful to compensate for already too high *p*-doping in some OFETs (often with a too positive *V_T_*).

It was actually quite surprising that even the strongest *p*-type doping at a full penetration depth only had a relatively small effect. At this point, we hypothesized that if the entire OFET becomes more severely contact limited [32], contact doping could manifest more benefits. Therefore, we prepared another undoped base structure with *E_b_* = 0.5 eV. This was performed by shifting the organic semiconductor HOMO position downward by 0.1 eV in the energy diagram (all the other parameters were kept the same). Before looking at doping effects, the undoped curve in Figure 3d clearly illustrates that a small increase in *E_b_* (from 0.4 to 0.5 eV) has brought a significant reduction in *I_D_*. Now, after introducing the *N*_contact-*p*_ of the same range to this modified base, it becomes obvious that such contact doping can actually be quite promising in overcoming a high contact resistance.

### 3.4. Comparative Analysis

**Figure 4:**
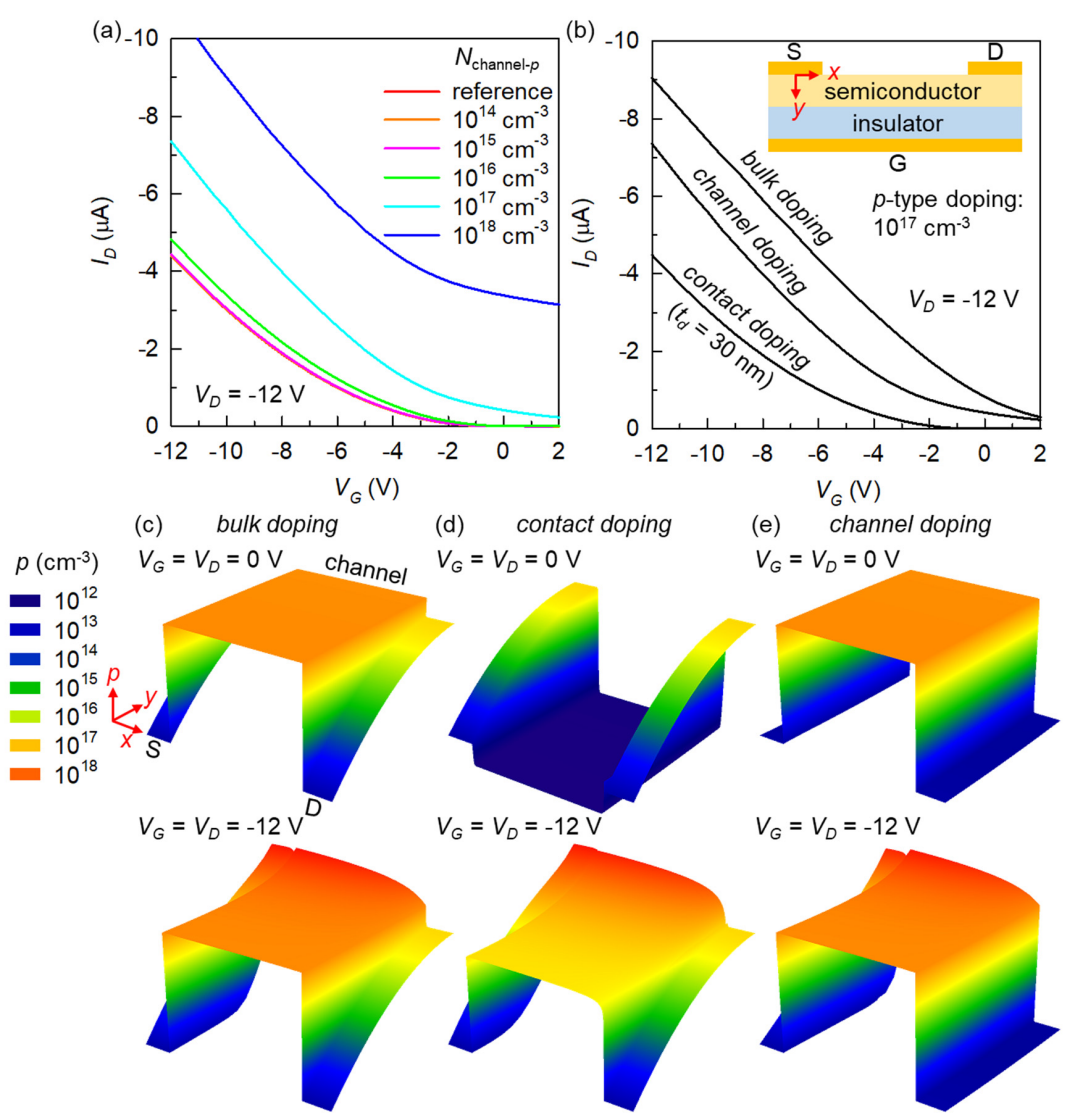
(**a**) Effects of selective channel doping on the OFET transfer characteristics. (**b**) Direct comparison between the three doping methods. Inset: the common device structure onto which the 2D positional axes are indicated. The 3D plots showing the characteristic in-semiconductor charge distribution for (**c**) bulk-doped, (**d**) contact-doped, and (**e**) channel-doped transistors. The *p*-type doping concentration is 10^17^ cm^−3^. Each of these panels contains the data for the thermal equilibrium (**top**) and fully-on conditions (**bottom**). The text guides for the positions of source, drain, and channel, as well as the 3D axis directions and color legend to the top part of panel (**c**) are common to all three panels (and six plots). The axes are intentionally not overlaid for the maximum focus on data surface shapes. To be precise, the *x* coordinate is from 0 to 70 μm (to include a 10-μm source and drain regions at both sides of the 50-μm channel), the *y* coordinate is from 0 to 30 nm (from the top semiconductor surface to the dielectric interface), and the *p* axis is in log scale from 10^12^ to 10^18^ cm^−3^.

The major motivation of this research is to draw a side-by-side comparison between bulk and contact doping as the two main techniques for OFETs. Based on the findings above, we found it interesting to add another distinct doping scheme called channel doping, which is characterized by the absence of doping beneath the source and drain electrodes (Figure 1d) [43]. This is mainly to extend the ideas of location control for doping and to further explore the unique mechanisms of doped OFETs. We therefore simulated selectively channel-doped transistors introducing a *p*-type channel doping density (*N*_channel-*p*_) from 10^14^ to 10^18^ cm^−3^. Figure 4a illustrates that, overall, channel doping can yield an intermediate result between those obtainable from bulk and contact doping systems. Here, we observe a doping-induced *I_D_* increase that is much more significant than that from contact doping (see in association with Figure 3b), yet the transistor off-state is still achievable even at a moderately high doping concentration (see in association with Figure 2b). For a direct comparison, we collected the transfer curves with a *p*-doping concentration of 10^17^ cm^−3^ in Figure 4b.

For comprehensive structural insights into doping methods, we finally present a series of three-dimensional (3D) solutions for the electrostatic distribution that most intuitively visualize the critical *x*-*y*-*p* relationship in the doped OFETs, as shown in Figure 4c–e. Here, the positional axes (*x* and *y*) should be referenced to the inset of Figure 4b. What is commonly important to a proper understanding of these results is that there are two critical boundary conditions that are not much affected by the existence and degree of charge doping. First, the *p* value right at the interface between a metal and semiconductor is solely determined by the *E_b_* = 0.4 eV (dictated by Boltzmann statistics) [18] and, therefore, serves as one fixed boundary. Returning to Figure 4c–e, this fixed boundary is why all six plots feature exactly the same *p* values at the source and drain contacts, which are of the order of 10^13^ cm^−3^ (it was actually already captured in Figure 2d). Second, the *p* value at the switched-on, accumulated channel bears its capacitive nature, so is (primarily) *V_G_*- and (laterally) *V_D_*- dependent. This explains the nearly identical pinched-off *p* profile near 10^18^ cm^−3^ for all three turned-on transistor channels (even including the completely undoped channel in the contact-doped OFET) in Figure 4c–e (bottom plots). Therefore, the actual charge distribution can be understood as an outcome of balancing between these two boundary values and the thermal carrier density of the semiconductor itself, which is directly affected by the material bandgap for an undoped region and by the doping concentration for a doped region.

In the bulk-doped OFET (Figure 4c), at *V_G_* = *V_D_* = 0 V, the semiconductor region between the source and drain contacts has a flat *p* surface directly reflecting an *N*_bulk-*p*_ of 10^17^ cm^−3^. In stark contrast, the regions below the electrodes are strongly depleted and, therefore, have orders-of-magnitude lower and vertically varying *p* values. The application of *V_G_* = *V_G_* = −12 V did not much change the overall shape, while the channel is slightly more accumulated. It therefore becomes clear why the on–off switching contrast (in terms of *I_D_*) is very low in the bulk-doped OFET, in that the external biasing does not greatly contribute to the film electrostatistics. In the case of contact doping (Figure 4d), an extremely low *p* is observed at the mid-semiconductor at zero biases. The exact value of the order of 10^12^ cm^−3^ is indicative of a bandgap as high as 3.3 eV (Table 1). The channel region is, therefore, apparently more depleted than the contact regions in this situation. By applying *V_G_* = *V_G_* = −12 V, the overall distribution becomes similar to that in the bulk-doped counterpart. However, we can recognize that the back-channel *p* (at the top semiconductor surface) is reduced because of both the absence of dopants and the screened gate-field effect there, which successfully rationalizes the lowest on-state *I_D_* among the three methods. The channel doping (Figure 4e) reveals globally intermediate features, thus rationalizing Figure 4b. Despite the *p* profiles (at both zero-biasing and turn-on) similar to those of the bulk doping, the organic regions under the source and drain electrodes are shown to be much more depleted than the bulk-doped case over the whole semiconductor thickness. Interestingly, the *V_G_* = *V_G_* = −12 V data here indicate that the high-*p* back channel can largely compensate for the source and drain regions that are more depleted than the contact-doped OFET, in improving the on-state *I_D_* levels.

## 4. Conclusions

We have investigated the bulk, contact, and channel doping concepts for OFETs. The use of physically based, finite-element simulation was key to unveiling the essential features of doped OFETs toward practical applications. The bulk doping method can be preferred for facile implementation (i.e., mixed solution casting and/or dopant deposition without spatial masking). Our results confirmed that it can most widely modulate *I_D_* and *V_T_*, while decoupling of these two seems difficult. Therefore, a density restricted to a certain level (which might be dependent on actual materials and device properties) should be desirable to build an improved transistor. Contact doping, on the other hand, did not strongly affect the OFET characteristics in our case. We found that this particular doping can be otherwise useful for improving contact-limited transistors due to a high *E_b_*. Therefore, this type of doping can be recommended to overcome a limited materials combination (it can be useful if we cannot reduce *E_b_* below a certain level), as it has an overall advantage of reduced dopant consumption and minimal interference with channel switching. According to our simulation, channel doping can provide a promising balance between these two techniques. It was shown to be effective in substantially increasing *I_D_*, while the *V_T_* shift remained in a realistic range owing to the contact depletion (therefore, the contact resistance provides a positive effect in this case [32]). Our simulation results were fully supported by an in-depth correlation between terminal characteristics and internal distribution, as well as extensive theoretical rationalization based on OFET device physics. Therefore, we believe that these results will significantly contribute to the development of next-generation, high-performance OFETs by delivering a set of practical design rules related to controlled doping methods and their electrical functionalities.

## Figures and Tables

**Table 1:** List of input parameters that produce the reference simulation curve in Figure 1a.

Layer	Parameter	Value
Organic semiconductor	Electron affinity	2.0 eV
	Band gap	3.3 eV
	Doping concentration	0 cm^−3^
	Hole mobility	1 cm^2^V^−1^s^−1^
	HOMO effective DOS	10^20^ cm^−3^
	Total trap density	10^17^ cm^−3^
	Trap characteristic temperature	1200 K
Gate electrode	Work function	5.0 eV
Source/drain electrodes	Work function	4.9 eV
